# Randomized controlled multicentre study comparing biological mesh closure of the pelvic floor with primary perineal wound closure after extralevator abdominoperineal resection for rectal cancer (BIOPEX-study)

**DOI:** 10.1186/1471-2482-14-58

**Published:** 2014-08-27

**Authors:** Gijsbert D Musters, Willem A Bemelman, Robbert JI Bosker, Jacobus WA Burger, Peter van Duijvendijk, Boudewijn van Etten, Anna AW van Geloven, Eelco JR de Graaf, Christiaan Hoff, Niels de Korte, Jeroen WA Leijtens, Harm JT Rutten, Baljit Singh, Anthony van de Ven, Ronald JCLM Vuylsteke, Johannes HW de wilt, Marcel GW Dijkgraaf, Pieter J Tanis

**Affiliations:** 1 Department of Surgery, Academic Medical Centre, University of Amsterdam, Post box 22660, Amsterdam 1105AZ, The Netherlands; 2 Department of Surgery, Deventer Hospital, Post Box 5001, Deventer 7400CC, The Netherlands; 3 Department of Surgery, Erasmus Medical Centre / Daniel den Hoed, Post box 2040 3000, Rotterdam, CA, The Netherlands; 4 Department of Surgery, Gelre Hospital, Albert Schweitzerlaan 31, Apeldoorn, DZ 7334, The Netherlands; 5 Department of Surgery, University Medical Centre Groningen, University of Groningen, Hanzeplein 1, Groningen, RB 9700, The Netherlands; 6 Department of Surgery, Tergooi Hospital, Post box 10016, Hilversum, DA 1201, The Netherlands; 7 Department of Surgery, IJsselland hospital, Post box 690, Capelle aan de Ijssel, AR 2900, The Netherlands; 8 Department of Surgery, Medical Centre Leeuwarden, Henri Dunantweg 2, LeeuwardenAD 8934, The Netherlands; 9 Department of Surgery, Spaarne Hospital, Spaarnepoort 1, Spaarne, TM 2134, The Netherlands; 10 Department of Surgery, St. Laurentius Hospital, Monseigneur Driessenstraat 6, Roermond, CV 6043, The Netherlands; 11 Department of Surgery, Catherina hospital, Eindhoven, EJ and Maastricht University Medical Centre, Michelangelolaan 2, P. Debyelaan 25 6229, Maastricht, HX 5623, The Netherlands; 12 Department of Surgery, Leicester Hospital, Gwendolen Rd, Leicester, UK; 13 Department of Surgery, Flevohospital, Hospitaalweg 1, Almere, RA 1315, The Netherlands; 14 Department of Surgery, Kennemer Gasthuis, Boerhaavelaan 22, Haarlem, RC 2035, The Netherlands; 15 Department of Surgery, Radboud University Medical Centre, Geert Grooteplein-Zuid 22, Nijmegen, GA 6525, The Netherlands; 16 Clinical Research Unit, Academic Medical Centre, University of Amsterdam, Post box 22660, Amsterdam 1105AZ, The Netherlands

**Keywords:** Abdominoperineal resection, Rectal cancer, Radiotherapy, Primary perineal wound closure, Biological mesh, perineal wound infection, Perineal wound healing

## Abstract

**Background:**

Primary perineal wound closure after conventional abdominoperineal resection (cAPR) for rectal cancer has been the standard of care for many years. Since the introduction of neo-adjuvant radiotherapy and the extralevator APR (eAPR), oncological outcome has been improved, but at the cost of increased rates of perineal wound healing problems and perineal hernia. This has progressively increased the use of biological meshes, although not supported by sufficient evidence. The aim of this study is to determine the effectiveness of pelvic floor reconstruction using a biological mesh after standardized eAPR with neo-adjuvant (chemo)radiotherapy compared to primary perineal wound closure.

**Methods/Design:**

In this multicentre randomized controlled trial, patients with a clinical diagnosis of primary rectal cancer who are scheduled for eAPR after neo-adjuvant (chemo)radiotherapy will be considered eligible. Exclusion criteria are prior radiotherapy, sacral resection above S4/S5, allergy to pig products or polysorbate, collagen disorders, and severe systemic diseases affecting wound healing, except for diabetes. After informed consent, 104 patients will be randomized between standard care using primary wound closure of the perineum and the experimental arm consisting of suturing a biological mesh derived from porcine dermis in the pelvic floor defect, followed by perineal closure similar to the control arm. Patients will be followed for one year after the intervention and outcome assessors and patients will be blinded for the study treatment. The primary endpoint is the percentage of uncomplicated perineal wound healing, defined as a Southampton wound score of less than II on day 30. Secondary endpoints are hospital stay, incidence of perineal hernia, quality of life, and costs.

**Discussion:**

The BIOPEX-study is the first randomized controlled multicentre study to determine the additive value of using a biological mesh for perineal wound closure after eAPR with neo-adjuvant radiotherapy compared to primary perineal wound closure with regard to perineal wound healing and the occurrence of perineal hernia.

**Trail registration number:**

NCT01927497 (Clinicaltrial.gov).

## Background

Conventional abdominoperineal resection (cAPR) for distal rectal cancer is associated with relatively high rates of positive resection margins and iatrogenic tumour perforation. Pooled analysis of five European randomized controlled trials revealed that cAPR was an independent risk factor for local recurrence (19.7% versus 11.4%) and associated with a decreased 5-year survival rate (59% versus 70%) compared to low anterior resection [1]. Following the total mesorectal excision (TME) plane all the way down to the pelvic floor, as performed in cAPR, results in the typical coning of the specimen and thereby compromising resection margins. Extralevator APR (eAPR) reduces the rate of positive resection margins and tumour perforation and improves oncological outcome based on a systematic review of non-randomized studies [2]. The extralevator approach entails en bloc resection of the distal rectum, sphincter complex and levator muscles. Besides a change in surgical technique, neo-adjuvant radiotherapy has also contributed to better locoregional control in patients with distal rectal cancer.

Both wider excisions as a result of eAPR as well as increased use of neo-adjuvant (chemo)radiotherapy have significantly increased perineal wound healing problems, which have been reported in up to 59% [3-6]. Furthermore, perineal hernia is more likely to occur with a reported incidence of up to 20% [7]. Perineal wound complications may only consist of superficial infection or minor dehiscence, but are often more serious with major dehiscence or deep abscesses. Impaired perineal wound healing is a significant clinical problem being associated with increased hospital stay, the need for re-operation, and intensive wound care for several months. There is even a small proportion of patients in whom a presacral or perineal sinus remains. Quality of life may be severely impaired because of pain, smelly wound discharge, and interference with basic daily activities such as sitting and walking.

Internationally there is a trend towards the use of additional closure techniques after eAPR in an attempt to overcome this clinical problem. One of these techniques is the reconstruction of the pelvic floor with a biological mesh, but available literature data are scarce. A recently published review included 12 studies, of which six were only conference abstracts, with total numbers of included patients between 2 and 24 [8]. The percentage of minor wound complications in series with at least 5 patients ranged between 17% and 63%, and of major complications between 9% and 27%. Two larger studies not included in this review reported a perineal wound complication rate of 50% and 11% in 30 and 35 patients respectively [9,10]. Autologous tissue flaps might also be an option and seems to be equally effective as a biological mesh [11]. However, the routine use of flaps after eAPR for rectal cancer is controversial. The rectus abdominis muscle (RAM) flap cannot be combined with a laparoscopic approach, will add donor site morbidity, substantially increases operating time and often implicates assistance by a plastic surgeon [12]. The gluteal flap does not disturb abdominal wall integrity, but seems not to prevent perineal hernia formation [7].

The popularity of the use of biological meshes for eAPR is probably the result of its successful application in repair of complex and infected hernias of the abdominal wall as well as for example pelvic organ prolapses [13,14]. However, the costs of a biological mesh ranges between 500 and 1800 euro per patient depending on the size of acellular porcine collagen implant and company. Based on the limited available data and costs of biological meshes, primary perineal wound closure after eAPR still remains the standard of care in many institutes. An unpublished survey among Dutch surgeons performed in 2012 revealed that 66% exclusively used primary perineal wound closure after APR, although the extralevator approach is not yet completely implemented at a national level. Using simple direct closure of the perineal wound after eAPR is supported by data from relatively large cohort series (104 to 282 patients), which reported perineal wound complication rates between 17% and 35%, with a proportion of patients who received radiotherapy between 31% and 60% [5,15-17]. However, standardized perineal wound assessment was not used in any of these studies and complication rates may be underestimated because perineal wound healing was not a primary outcome parameter. This underlines the need for a randomized comparison of different perineal closure techniques after eAPR.

## Methods/Design

### Objective

The primary objective of this study is to determine the effectiveness of pelvic floor reconstruction using a biological mesh after eAPR with neo-adjuvant radiotherapy compared to primary perineal closure. It is hypothesized that biological mesh closure is superior to primary perineal closure in a cost-effective manner in terms of a higher primary perineal wound healing rate and a lower perineal hernia rate at reduced costs.

### Design

**Figure 1:**
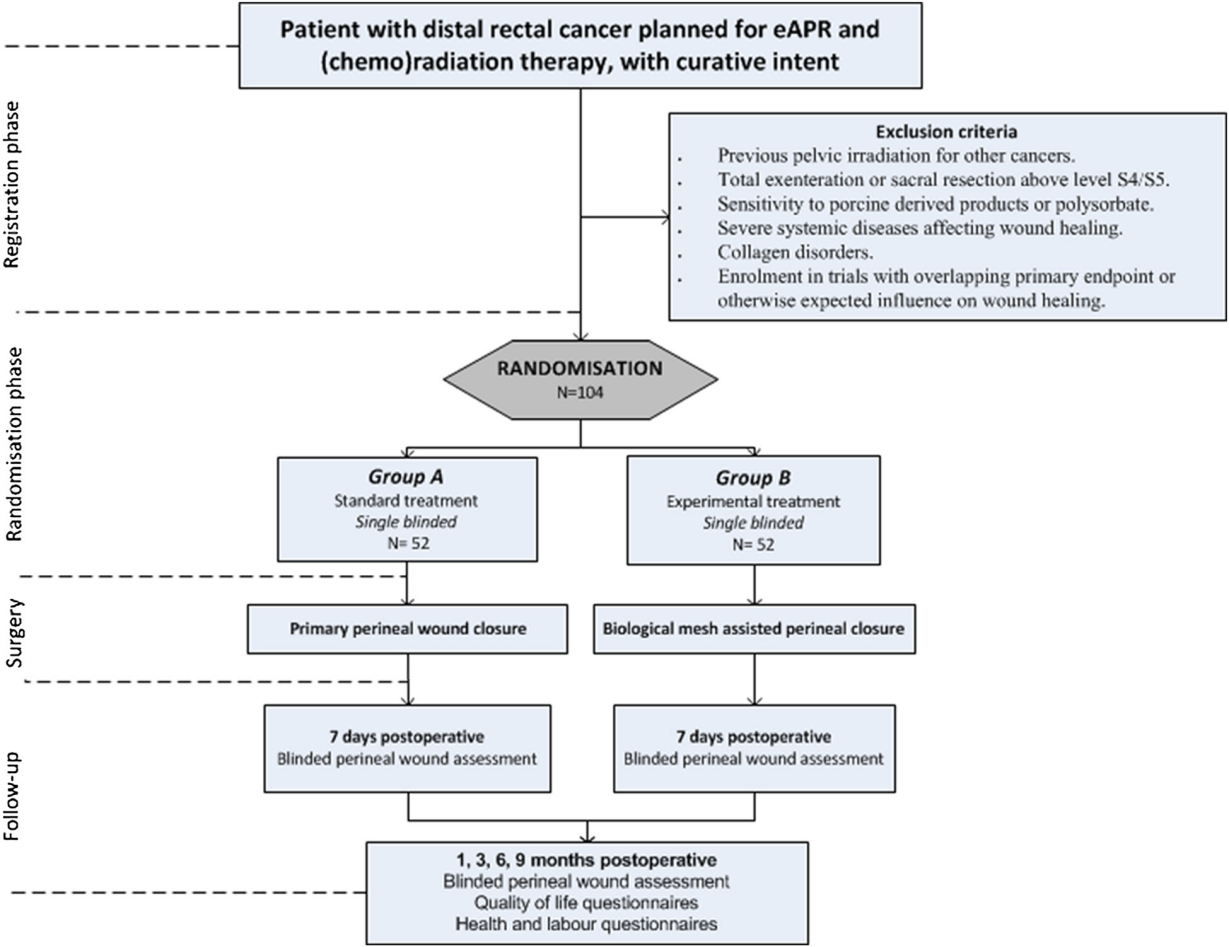
**Flow-diagram.** eAPR: abdominal perineal resection.

This is a multicentre, single blinded, randomized controlled trial, in which eligible patients will be randomized between pelvic floor reconstruction using a biological mesh (intervention arm) and primary closure of the perineal defect (standard arm) in a 1:1 ratio (Figure 1). Randomisation will be performed pre-operatively by a central automated randomisation using the trial website, with stratification for age, sex and laparoscopic surgery. The allocation of treatment is blinded to the patient.

The trial will be conducted in five academic centres and eight teaching hospitals, including two national referral centres for locally advanced rectal cancer. Participating centres will perform standardized perineal dissection according to the extralevator approach. The procedures in the experimental arm in centres without experience in biological mesh reconstruction of the pelvic floor will be supervised by one of the principal investigators (PT and WB) until a standardized biological mesh placement is ensured. Furthermore, a preceding investigators’ meeting will be organized at the start of the trial and a refreshment course during the inclusion period, both including a workshop using fresh frozen cadavers. The perineal wound healing will be evaluated at 7 and 30 days postoperatively using the Southampton wound scoring system by an independent nurse or physician not aware of the intervention to which the patient was allocated [18]. In addition, full colour photographs will be taken from the perineal wound and assessed by an expert panel (PT, WB) blinded for treatment allocation. During routine outpatient clinic visits for oncological follow-up at 3, 6, 9 and 12 months, the perineal wound will be inspected and scored accordingly with respect to healing and herniation (see Figure 1). In addition, CT-scan of the pelvis as usually performed during oncological follow-up at 1 year, will be reviewed with respect to presacral or perineal sinuses and perineal herniation. Quality of Life questionnaires will be administered to the patient at each follow-up interval. Patients without primary perineal wound healing who are treated in a non-hospital setting will get a wound diary in which the number of wound dressing changes, the number of visits by a home care nurse if applicable, and the wound materials used are registered per week until final closure of the perineal wound or end of study period. In addition, the nature and severity of any wound event, all medical or surgical interventions and re-admissions will be collected.

### Study population

Patients are eligible for this study when they meet the following inclusion criteria: age of 18 years or older, planned eAPR for primary rectal cancer, pre-operative (chemo)radiotherapy, life expectancy of more than 2 years, ability to return for all scheduled and required study visits, and written informed consent for study participation. Exclusion criteria are: previous pelvic irradiation for other cancers (i.e. prostate cancer), total pelvic exenteration or sacral resection above level S4/S5, sensitivity to porcine derived products or polysorbate, severe systemic diseases abibuffecting wound healing except diabetes (i.e. renal failure requiring dialysis, liver cirrhosis, and immune compromised status like HIV), collagen disorders (i.e. Marfan), enrolment in trials with overlapping primary endpoint or otherwise expected influence on wound healing (i.e. systemic therapy like anti-angiogenic agents). An interim review will be performed at 50 included patients (of the total of 104 patients). At 6 weeks after inclusion of these patients, the trial’s safety data will be evaluated. The steering committee will be supplied with the number of (serious) adverse events in both groups at this time point. If there is a skewed distribution of the number of (serious) adverse events between the two groups, an efficacy analysis can be performed at the discretion of the steering committee. Following these interim analyses, the steering committee will advise upon continuation of the trial.

### Intervention strategies

The perineal phase of the APR will be performed according to the principles of an extralevator approach, which means that the levator muscles will be laterally transected in order to leave a muscular cuff around the tumour. The coccyx will not be routinely resected, but only if indicated based on surgical exposure or oncological principles. The extent of excision of perineal skin and ischioanal fat will be as limited as oncologically justified. The eAPR specimens will be classified according to Phil Quirke and photographed in a standardized fashion. Patient positioning (prone or supine), the surgical approach for the abdominal phase (open or laparoscopic), and the use of an omental plasty are left to the discretion of the operating surgeon, because there is no conclusive evidence on which of these technical aspects is the most optimal with regard to perineal wound healing.

Closure of the perineum in the control arm consists of stitching the ischioanal and subcutaneous fat using interrupted Vicryl sutures in one or two layers. Subsequently, the skin will be closed using interrupted sutures according to the preference of the surgeon. Placement of a transabdominal or transperineal drain will be performed according to local protocols.

The intervention in the experimental arm consists of suturing an acellular biological mesh derived from porcine dermis in the pelvic floor defect (Strattice™, 6x10 cm). The mesh will be sutured at each side of the coccyx or distal sacrum with Prolene or Polydioxanone sutures (PDS). Laterally, the mesh is attached to the remaining of the levator complex and, anteriorly, to the transverse perineal muscle. A suction drain will be inserted and positioned on top of the mesh. The perineal subcutaneous fat and skin will be subsequently closed in layers similar to primary perineal closure as performed in the standard arm.

### Outcome parameters

Primary endpoint is the percentage of uncomplicated perineal wound healing defined as a Southampton wound score less than II at 30 days postoperatively.

Secondary endpoints of this study are the need for wound care, either in hospital or out of hospital and specified to frequency, duration, and type of wound dressing, the need for surgical re-intervention or re-admission for perineal wound problems, total hospital stay during one year of follow-up, incidence of asymptomatic and symptomatic perineal hernia, quality of life (EQ-5D-5 L, EORTC-30, EORTC-29RC, SF-36), and (in)direct medical and non-medical costs (clinical report forms, hospital information systems, modified health and labour questionnaire). In addition, perineal wound healing according to the Southampton wound grading will be repeated at 3, 6, 9 and 12 months postoperatively in order to determine differences in wound healing between the study arms over time. Differences in the incidence of persistent perineal or presacral sinuses, both clinically and by CT imaging, will be evaluated at 1 year of follow-up.

### Sample size calculation

The principal analysis will consist of an intention-to-treat comparison of the proportions of patients with primary uncomplicated perineal wound healing between both study arms. The hypothesis is to test superiority of the biological mesh assisted closure over primary closure of the perineum. The currently available literature is difficult to interpret with regard to perineal wound healing for the two interventions to be studied, hampering defining the expected difference in healing rate. Data on primary closure are mostly derived from cohort series in which patients were mostly operated upon before 2005 [3-7,10,19-23]. At that time, eAPR was only applied in a minority of centres. Furthermore, there has been an enormous increase in the use of neo-adjuvant radiotherapy in the past decade. These two developments in the treatment of distal rectal cancer have had a significant impact on perineal healing rate. In contrast, literature on biological mesh assisted perineal closure has been published recently only in patients undergoing eAPR and with high rates of neo-adjuvant radiotherapy [7,9,10,20-23]. In addition, these studies had a specific focus on perineal wound healing, which results in more detailed data with a relative over-reporting of perineal wound complications compared to the historical data on primary closure in which perineal healing was most often a secondary endpoint that was retrospectively studied. Both the low percentage of eAPR in the primary closure group and relative over-reporting of perineal wound complications in the biological mesh led us to conclude that the available literature may underestimate the potential difference between both closure techniques.

Given the before mentioned lack of high quality data in the literature, we defined a clinically relevant difference in primary uncomplicated perineal healing which justifies the routine use of an expensive biological implant. This difference is considered to be 25%. A total number of 98 patients (49 per group) are needed to be able to detect a 25% increase in primary perineal wound healing by insertion of a biological mesh from 60% to 85%, applying a Chi2-test with a two-sided 0.05 significance level and with 80% power. With an estimated drop-out of 5%, a total number of 104 patients are required (52 per group).

### Data-analysis

The primary endpoint, the percentage of uncomplicated wound healing defined as a Southampton wound score of less than II at 30 days postoperatively, will be compared between the two study groups using a two-sided Chi-square test with a significance level of 0.05. The primary endpoint will be further explored by comparing wound scores as categorical variable using the Mann Whitney U test. Furthermore, differences in time to healing between the two groups will be analysed as a censored continuous variable using Kaplan-Meier survival analysis. Treatment effects will be expressed as a relative risk with 95% confidence interval. Any binary secondary outcome measures (e.g. hernia rate, re-operation rate, infection rates, etc.) will be analysed in the same way as the primary outcome. Quality of life data (e.g. EQ-5D-5 L, EORTC-30, EORTC-29RC, SF-36) will be graphically represented across all time points and analysed using a repeated measures analysis of variance. The scoring profiles from a patient on subsequent EQ-5D-5 L assessments up to month 12 will further be used to derive health utilities based on the most recent crosswalk value sets at the time of analysis [24]. The health utilities will then be used to calculate quality adjusted life years (QALY) as the product sum of the utilities and the lengths of time preceding the repeated measurements.

The most recent Dutch guideline for costing in health care research will be applied to assign unit costs to observed health care resources in order to estimate the costs of care for each study group [25]. Costs differences will be off-set against the difference in numbers of patients with uncomplicated perineal wound healing. Cost differences will also be off-set against the difference in quality adjusted life years to calculate the extra costs per QALY gained. Both economic analyses will be performed from a societal perspective, with a time horizon set at 12 months. In addition, the analyses will be repeated from a hospital care provider perspective to improve the study’s generalizability to countries with other health care systems. No discounting of effects and costs will be performed. Sensitivity analyses will be performed to account for sampling variability and key study parameters (unit costs of the closure procedure using the biological mesh, alternative utility weights of observed health states). Results will be displayed graphically with cost-effectiveness planes and acceptability curves.

### Ethics and safety

The medical ethical committee of the Academic Medical Centre, Amsterdam, the Netherlands, has approved the study protocol (MEC 2012–360, NL42094.018.12). This study will be conducted according to the principles of Good Clinical Practice.

## Discussion

The BIOPEX study is the first randomized controlled trial comparing a new perineal closure technique after eAPR with primary perineal closure. There is one other registered ongoing trial on perineal closure after eAPR, but not including a study arm consisting of what we consider to be the standard of care, namely primary wound closure. This is a Swedish study randomizing between gluteus maximus myocutaneous flap and acellular porcine collagen implant (NEAPE; trials.gov identifier NCT01347697). Pelvic floor reconstruction using a musculocutaneous flap such as the RAM flap is often indicated in large irradiated perineal defects, for example after salvage surgery for residual or recurrent anal cancer [26-28]. But routinely using a musculocutaneous flap for perineal reconstruction after APR for rectal cancer, except for exenterative procedures, seems to be over-treatment [12]. The gluteal flap as used in the Swedish trial does not disturb abdominal wall integrity, but seems not so attractive by significantly increasing surgical trauma in the irradiated perineum with postoperative mobilisation problems. If the gluteal flap is used as a fasciocutaneous flap, it is associated with an unacceptably high rate of perineal hernia development according to Christensen et al. [7]. The biological mesh has been recently introduced and appears to be a valid alternative for the autologous tissue flaps [7,9,10,19-23]. Since the perineal wound is by definition contaminated, a biological mesh, being more resistant to infection, is preferred over less expensive synthetic meshes. Clinical outcome of a biological mesh appeared to be comparable to flap assisted perineal closure in a non-randomized comparison based on systematic review of the literature [11]. Furthermore, a biological mesh is associated with lower costs per patient compared to a RAM flap [21]. Others have suggested an omental plasty for perineal reconstruction, but this functions as filling of dead space rather than giving strength to a neo-pelvic floor. A biological mesh differs essentially from a tissue flap by not filling the pelvic defect. Combining an omental plasty with a biological mesh may therefore be of additive value. Although a high level of evidence is lacking, an omental plasty is recommended after APR, because it adds well vascularised, non-irradiated tissue to the pelvic cavity [16]. Omental plasty is not routine care in some of the participating centres of the BIOPEX study, which is the reason that we decided not to include this into the study protocol as a required part of the reconstruction.

The costs associated with routinely adding a biological mesh to the surgical procedure is supposed to ultimately translate into cost saving by reducing hospital stay, reducing the need for operative reintervention, reducing the need for intensive wound care and reducing costs related to secondary repair of perineal hernia. There is even a subgroup of patients with a persisting perineal sinus after one year that is unlikely to heal eventually [29]. Non-healing at one year after pre-operative radiotherapy has been reported in up to 26% and may also be reduced by biological mesh assisted closure [19]. The percentage of surgical reintervention for perineal complications is reported between 8% and 16% and is expected to be halved from 10% to 5% by the use of a biological mesh [3,19,26]. Furthermore, it is expected that perineal hernia incidence will be reduced by at least 10%, based on a difference of 20% as recently published by Christensen et al. [7]. Although this was a comparison between gluteal flap and biological mesh, gluteal flap is similar to primary closure regarding risk of perineal hernia because it does not reconstruct the pelvic floor. Primary use of a biological mesh is therefore expected to reduce by more than half the costs related to secondary perineal hernia repair requiring hospital admittance for about three days with operative intervention.

In conclusion, pelvic floor reconstruction using biological meshes has been suggested to improve perineal wound healing and may potentially save net health care costs compared to simple primary closure of the perineum after eAPR, but proof of which is urgently needed.

## Abbreviations

BIOPEX: *Bio*logical mesh closure of the *p*elvic floor after *ex*tralevator abdominoperineal resection for rectal cancer; TME: Total mesorectal excision; cAPR: Conventional abdominoperineal resection; eAPR: Extralevator abdominoperineal resection; S4/S5: 4^th^ / 5^th^ sacral vertebra; PDS: Polydioxanone sutures; EQ 5D-5 L (Euroqol): this questionnaire is a simple, Generic instrument for describing and valuing health related quality of life. It includes 5 items (mobility, personal care, daily activities, pain, and anxiety-depression) that are answered on a 3-point scale ranging from no problems (level 1) to extreme problems (level 3). The health valuations are used together with the times spent in those health states to derive quality adjusted life years (QALY); Global quality of life (EORTC-QLQ-C30-QL2): This sub questionnaire contains the 2 items of the global quality of life dimension of the EORTC-QLQ-C30 questionnaire; Global quality of life (EORTC-QLQ-CR29): This questionnaire is developed to assess the quality of life in colorectal patients; Health and Labour questionnaire (adapted to the study setting): This questionnaire is designed to collect quantitative data on the impact of (treatment for) illness and resource utilization and absenteeism from work; Quality of Life questionnaire (SF36): This questionnaire measures the relationship between a client’s quality of life and other behaviours or afflictions; RAM: Rectus abdominis muscle flap.

## Competing interests

Three investigators (PT ,WB and BS) have been paid by LifeCell for giving lectures at symposia and congresses. The biological meshes are provided free of charge by the company LifeCell and they provided a start-up grant of €10,000.-. There are no other competing interests to be declared by any of the authors.

## Authors’ contributions

All authors have made substantial contributions to the conception and design of this study; have been involved in drafting the manuscript (GM, PT, MD, HR and WB) or revising it critically for important intellectual content and have given final approval of the version to be published. RB, PB, BE, PD, EG, NG, PH, CH, JL, NK, BS, RV, JW, and EZ have made contributions to the design of this study and have made substantial contributions to the organization of this trial. All authors have given final approval of the version to be published; and are local investigators at the participating centres.

## Pre-publication history

The pre-publication history for this paper can be accessed here:

http://www.biomedcentral.com/1471-2482/14/58/prepub
